# Investigating reasoning with multiple integrated neuroscientific methods

**DOI:** 10.3389/fnhum.2015.00041

**Published:** 2015-02-03

**Authors:** Matthew E. Roser, Jonathan St. B. T. Evans, Nicolas A. McNair, Giorgio Fuggetta, Simon J. Handley, Lauren S. Carroll, Dries Trippas

**Affiliations:** ^1^School of Psychology, Plymouth UniversityPlymouth, UK; ^2^School of Psychology, The University of SydneySydney, NSW, Australia; ^3^School of Psychology, The University of LeicesterLeicester, UK; ^4^Center for Adaptive Rationality, Max Planck Institute for Human DevelopmentBerlin, Germany

**Keywords:** reasoning, neuroscience methods, methodological integration, brain connectivity, neuronavigated TMS

Recent years have seen increased application of functional MRI (fMRI), transcranial magnetic stimulation (TMS) and event-related potentials (ERP), to questions of human rationality. This has both illuminated the brain bases of these functions and contributed to theoretical advances (Goel, 2007; Prado et al., 2008). Most studies have, however, employed only one method and the developing literatures run somewhat parallel with only informal integration of results across methods. Results from other fields (Sarfeld et al., 2012) demonstrate the potential benefits of integration of multiple neuroscientific methods within studies of human reasoning, allowing findings from one method to influence the application of other methods, or constrain the interpretation of data derived therefrom. Including data on regional brain volume, structural and functional connectivity, individual differences and development and aging is particularly appropriate to the study of neural mechanisms of human reasoning, which are likely to be formed from networks of numerous widely-distributed brain regions. Here we briefly describe how the integration of several neuroscientific methods within a single study may advance investigations of the reasoning brain.

fMRI has now been applied to a large number of reasoning paradigms (Goel, 2007). Consideration of what appears initially as a disparate set of brain activations reveals consistencies suggestive of several underlying neural systems. A formal analysis (Prado et al., 2011) of 28 studies found similar consistency of activation across studies and reasoning paradigms, but no monolithic neural system for reasoning. Instead, a collection of subsystems incorporating widely distributed areas of the brain is apparent. This widespread activation, encompassing frontal and posterior areas, in response to high-level tasks with long processing times complicates interpretation.

Approaches which move beyond mapping the spatial extent of activation to consider the quality of brain activity seen in separate regions promise to clarify the distributed-network nature of the reasoning brain. Analyses may focus on the time-courses of activation within brain regions (Rodriguez-Moreno and Hirsch, 2009), identifying subsets of regions involved at different stages of reasoning, or, as in our current research (ESRC Grant RES-062-23-3285), the correlation in the degree of activation seen in separate clusters with individual differences (Reverberi et al., 2012).

Formal analyses of functional connectivity, or correlated activity (Friston, 2011), between brain regions active during the resting state have revealed the effects of prolonged practice on a reasoning task (Mackey et al., 2013). The application of functional-connectivity analyses to brain activity elicited by reasoning, rather than rest, awaits. While many imaging studies of reasoning speak of the “networks” involved it would be more accurate to speak of distributed regions of task-related activation as no studies have formally tested functional connectivity between regions. This is in contrast to other areas, such as research in memory, attention and task control, in which functional-connectivity analyses are commonplace and have greatly advanced the characterization of implicated brain networks (Vincent et al., 2008). Functional-connectivity analyses have the potential to further clarify how subsets of the numerous regions found active in fMRI studies of reasoning group together to form dynamic networks that are reconfigured across extended periods of reasoning-task performance.

A further step is analysis of effective connectivity in which causal networks of distributed regions are modeled and tested against observed data (Friston, 2011). Models incorporate information about brain *structural* connectivity into predictions of inter-regional *functional* connectivity. These structural data have traditionally come from monkey section studies but human diffusion-tensor imaging (DTI) data are now being used, as described in a recent survey of methods and applications for fusing fMRI and DTI data (Zhu et al., 2014). DTI is a MRI technique which allows the microstructural connectivity of brain tissue to be probed (Le Bihan, 2003). The data can be acquired in a scan lasting only around 10 min, which could feasibly be included in a fMRI study. DTI data have informed researchers about the constraining effect of structural connectivity upon functional connectivity in non-reasoning tasks (Honey et al., 2009). Structural-connectivity maps of direct and indirect connections between brain regions were tested as predictors of resting-state inter-regional functional connectivity, leading to a model in which functional connectivity is determined by a combination of direct and indirect structural connections. Ultimately, the integration of fMRI and DTI datasets could allow the development of richer models of dynamic networks of distributed brain regions supporting reasoning performance. Putative networks of brain regions activated by reasoning tasks may be merely regions of correlated activity that do not exist in a causative relationship, or they may be comprised of two or more overlapping and commonly-activated sub-networks. These possibilities can be tested using models informed by integrated methods.

The further integration of a developmental or aging perspective, to which DTI is sensitive (Sullivan and Pfefferbaum, 2006), would allow the organization and degeneration of brain structural connectivity, and its role in supporting reasoning, to be traced over the lifespan. Information on brain regional and connective development and degeneration is of great relevance to a growing literature (Salthouse, 2005) of age effects on reasoning. The anterior to posterior progression of degeneration in the aged brain, apparent in DTI studies (Sullivan and Pfefferbaum, 2006), predicts that reasoning processes that draw heavily on frontal support will be more affected by age than are reasoning processes that primarily involve posterior regions. Also of relevance is information about brain regional volume, as assessed by MRI, which has been shown to be abnormal in some populations, such as people with autism (Mcalonan et al., 2005; Redcay and Courchesne, 2005), who are also of interest to investigators of reasoning (Mckenzie et al., 2010; Morsanyi and Holyoak, 2010).

The incorporation of structural and functional MRI into studies of reasoning using repetitive TMS has promise to increase the power and accuracy of a technique which can probe the causal relationship between brain activity and reasoning performance. Previous rTMS studies (Tsujii et al., 2010, 2011) guided stimulation using structural MRI but selected cortical targets somewhat arbitrarily from a set of areas implicated in fMRI studies. An improvement is to integrate results from an fMRI study using the same paradigm and stimuli to target specific locations found to be functionally active. As considerable variation in reasoning-associated activation across studies using similar, but non-identical, paradigms, and stimuli has been observed (Goel, 2007) the targeting of specific areas activated by specific experimental designs is important. We (ESRC Grant RES-062-23-3285) are doing this by warping the standard-space group-analysis results from our fMRI study of conditional reasoning into the individual TMS-subject space to identify functionally-relevant targets. Furthermore, using a within-trial, short-burst rTMS paradigm (Fuggetta et al., 2008), allows greater temporal specificity in rTMS application. By disrupting activity in ventral and dorsal prefrontal cortex at different stages of conditional-reasoning trials we predict a double dissociation of the effect of rTMS on belief bias at the two locations over the two stages of the trial. This result would advance our understanding of the processes involved in conditional reasoning, and of the roles of the two brain regions, and is an example of how method integration might inform psychological theory.

ERP studies of reasoning differ in the degree to which they preserve the traditional behavioral paradigms (Qiu et al., 2009; Luo et al., 2013), which typically involve extended reading, and the temporal specificity with which they are able to resolve reasoning processes by adapting orthodox paradigms shown to elicit well-defined ERPs (Prado et al., 2008; Banks and Hope, 2014). Despite this heterogeneity, evidence is accumulating that ERPs and oscillatory activity associated with expectation and inhibition are modulated by performance on reasoning tasks (Bonnefond and Van der Henst, 2009; Bonnefond et al., 2014). Initial steps to identify the neural sources of observed ERPs (Qiu et al., 2009; Luo et al., 2013) could be greatly improved by using results from fMRI studies to constrain the fitting of source models. The ultimate aim is to conduct simultaneous recordings of EEG and fMRI (Baumeister et al., 2014), illuminating sequential activations across distributed networks, as are revealed by the less-available technique of magnetoencephalography (Bonnefond et al., 2013).

A full characterization of the reasoning brain will require models that describe functional connectivity between widespread brain regions, constrained and shaped by structural connectivity, which varies between and within individuals across time and space. This implies a conceptualization of the reasoning brain as a spatially-extended dynamical system. Models of this type will necessarily integrate data derived from many different methods and may require mathematical tools not previously applied to investigations of reasoning (Siegelmann, 2010). At present most of these techniques are being applied to the study of the reasoning brain, but in a parallel fashion. The lesson from other areas of investigation (Calhoun and Lemieux, 2014) is that their integration can yield more than the sum of their parts.

## Conflict of interest statement

The authors declare that the research was conducted in the absence of any commercial or financial relationships that could be construed as a potential conflict of interest.
